# Chronic Model of Inflammatory Bowel Disease in IL-10^-/-^ Transgenic Mice: Evaluation with Ultrasound Molecular Imaging

**DOI:** 10.7150/thno.37397

**Published:** 2019-08-14

**Authors:** Huaijun Wang, Jose G Vilches-Moure, Samir Cherkaoui, Isabelle Tardy, Charline Alleaume, Thierry Bettinger, Amelie Lutz, Ramasamy Paulmurugan

**Affiliations:** 1 Department of Radiology, Stanford University, School of Medicine, Stanford, California, USA; 2 Department of Comparative Medicine, Stanford University, Stanford, California, USA; 3 Bracco Suisse SA, Geneva, Switzerland

**Keywords:** inflammatory bowel disease (IBD), ultrasound molecular imaging (USMI), P-selectin, chronic disease, mouse model, Interleukin 10 deficient (IL-10^-/-^) mice, FVB mice, piroxicam, 2,4,6-trinitrobenzenesulfonic acid (TNBS), dextran sulfate sodium (DSS)

## Abstract

**Objective**: Acute mouse models of inflammatory bowel disease (IBD) fail to mirror the chronic nature of IBD in patients. We sought to develop a chronic mouse IBD model for assessing long-term anti-inflammatory effects with ultrasound molecular imaging (USMI) by using dual P- and E-selectin targeted microbubbles (MB_Selectin_).

**Materials and Methods**: Interleukin 10 deficient (IL-10^-/-^ on a C57BL/6 genetic background; n=55) and FVB (n=16) mice were used. In IL-10^-/-^mice, various experimental regimens including piroxicam, 2,4,6-trinitrobenzenesulfonic acid (TNBS) or dextran sulfate sodium (DSS), respectively were used for promoting colitis; colitis was induced with DSS in FVB mice. Using clinical and small animal ultrasound scanners, evolution of inflammation in proximal, middle and distal colon, was monitored with USMI by using MB_Selectin_ at multiple time points. Imaged colon segments were analyzed *ex vivo* for inflammatory changes on H&E staining and for P-selectin expression on immunofluorescence staining.

**Results**: Sustained colitis was not detected with USMI in IL-10^-/-^ or FVB mice with various experimental regimens. USMI signals either gradually decreased after the colitis enhancing/inducing drug/agents were discontinued, or the mortality rate of mice was high. Inflammation was observed on H&E staining in IL-10^-/-^ mice with piroxicam promotion, while stable overexpression of P-selectin was not found on immunofluorescence staining in the same mice.

**Conclusion**: Sustained colitis in IL-10^-/-^ mice induced with piroxicam, TNBS or DSS, and in FVB mice induced with DSS, was not detected with USMI using MB_Selectin_, and this was verified by immunofluorescence staining for inflammation marker P-selectin. Thus, these models may not be appropriate for long-term monitoring of chronic colitis and subsequent treatment response with dual-selectin targeted USMI.

## Introduction

Inflammatory bowel disease (IBD) is a chronic relapsing-remitting inflammatory disorder of the gastrointestinal tract. IBD includes two major phenotypes, Crohn's disease (CD) and ulcerative colitis (UC).

The incidence of IBD increased in Europe and USA in the last half of the century 1, 2, especially in the pediatric populations 3. In developing counties, the incidence of IBD is still comparatively low, but it has recently been shown that the incidence of IBD is also rapidly growing in these counties 4. In clinical practice, the diagnosis of IBD is mainly based on clinical manifestations and laboratory tests. However, such test findings do not correlate well with the physiological activity of IBD 5, 6. During the often life-long course of IBD, non-invasive and quantitative tools are necessary for the serial follow-up exams to monitor the disease activity and to evaluate treatment efficacy.

Transabdominal ultrasound imaging has a lot of advantages and is an ideal imaging tool for assessing various abdominal organs, for determining disease severity, and for monitoring therapeutic outcomes 7. Ultrasound imaging is relatively inexpensive, portable, radiation-free, available all over the world, and provides real-time images with high resolution 8. Contrast-enhanced ultrasound (CEUS) imaging with non-targeted microbubbles enables the assessment of vascularity of inflamed bowel wall and has been used in differentiating responders from non-responders of IBD patients under treatment 9-12. However, one of the disadvantages of CEUS is that only one bowel segment can be imaged during the injection of contrast microbubbles. Since multiple bowel segments are usually involved in IBD, sampling efficacy of IBD lesions is limited with CEUS using non-targeted microbubbles 13, 14.

Ultrasound molecular imaging (USMI) using molecularly targeted contrast microbubbles, addressing molecular markers of inflammation, such as P- and E-selectin, namely MB_Selectin_, provides the quantitative readout of inflammatory activity of IBD at the molecular level. Once intravenously injected, MB_Selectin_ remains attached to P- and E-selectin expressed in the inflamed vascular wall for an extended period of time, during which multiple bowel segments can be imaged. Dual-selectin targeted USMI has been validated in murine and swine models of IBD for assessing different degrees of inflammation and monitoring treatment outcomes using histology and immunofluorescence staining as gold standards 15-18. In these small and large animal models of IBD, acute inflammation has been chemically induced using [2,4,6-trinitrobenzenesulfonic acid (TNBS)]. These acute inflammation models are relatively easy to induce and thus have been frequently used in IBD imaging studies 15-18. However, TNBS-induced IBD is self-limiting and the inflammation spontaneously resolves within a few days, even without anti-inflammatory drug treatment. Due to the short duration of inflammation in those models, treatment outcomes can only be assessed during a short time window 18, 19. Therefore, these acute TNBS-induced IBD models fail to mirror the chronic course of IBD in patients and are not suitable for monitoring the effects of long-term treatment lasting weeks to months.

Genetically engineered models of IBD have been used for studying chronic intestinal inflammation for decades. Out of these models, the interleukin-10 deficient (IL-10^-/-^) mouse is one of the most frequently used models for studying IBD 20-22. IL-10 operates on innate and adaptive immune mechanisms to suppress inflammatory responses in the intestine. IL-10^-/-^ mice spontaneously develop IBD starting at 2-3 months of age, but only less than 5% of mice can develop spontaneous IBD between 2-8 months. It has been shown that the exposure of IL-10^-/-^ mice to piroxicam can result in rapid and uniform development of colitis, which persists for a long period of time even after piroxicam is discontinued 22-24. The histology of colitis in piroxicam-treated animals was reported to be similar to that observed when IL-10^-/-^ mice spontaneously develop IBD, and ultimately to human IBD 22, 24. Dextran sulfate sodium (DSS) induced colitis is another well-established animal model, which can be acute or chronic depending on the DSS dosage administered 25.

To our knowledge, there has been only one study where spontaneous chronic ileitis in a transgenic mouse model of IBD (SMAP) was evaluated by using USMI with mucosal addressin cellular adhesion molecule (MAdCAM)-1 targeted microbubbles 26. The purpose of current study was to develop a chronic mouse IBD model of for assessing long-term anti-inflammatory effects by using dual-selectin-targeted ultrasound molecular imaging.

## Materials and Methods

### Animals

**Figure 1:**
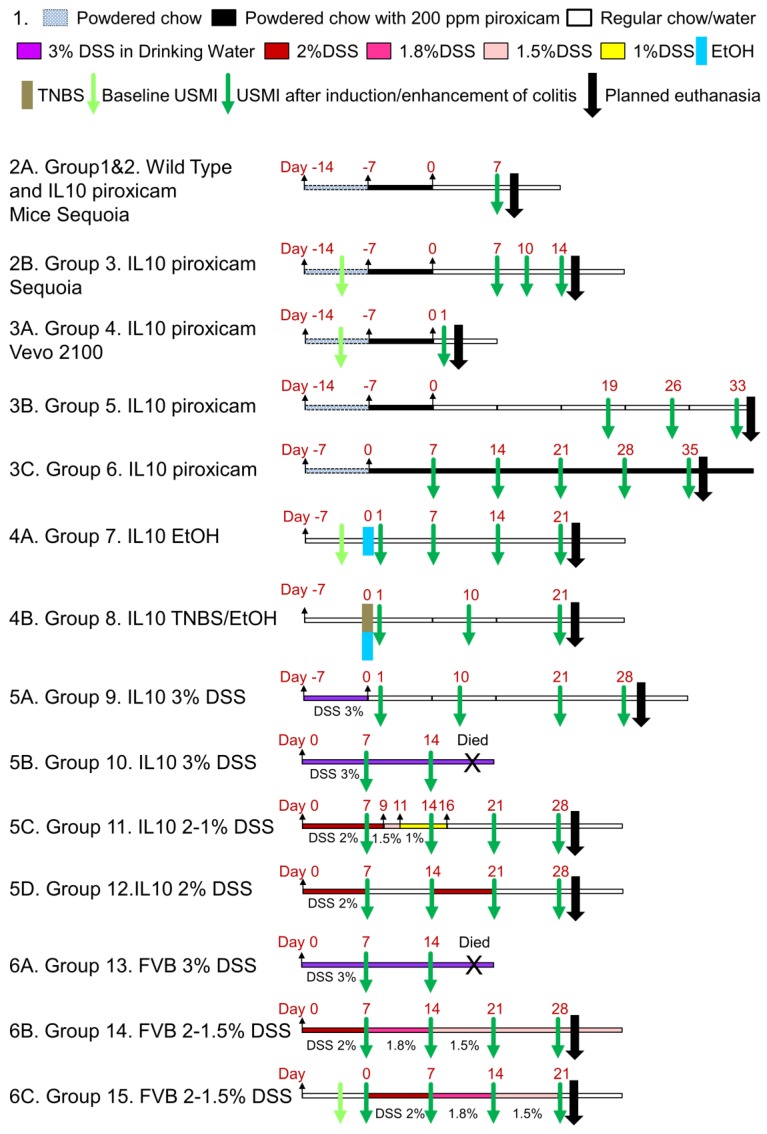
Overall experimental design.

The Institutional Administrative Panel on Laboratory Animal Care approved all procedures involving laboratory animals. Mice were purchased from Jackson Laboratories (Bar Harbor, ME). Commercially available female C57BL/6 IL-10^-/-^ mice (B6.129P2-Il10^tm1Cgn^/J; Stock No: 002251; 8-12 weeks old, n=55), female wild type C57BL/6 mice (6-8 weeks old, n=5), and female FVB mice (6-8 weeks old, n=16) were used in this study (total n=76). In this study, we explored different chronic colitis models by inducing/enhancing inflammation using various drugs/chemicals in different groups of mice as detailed in **Table 1**. The experimental design is summarized in **Figure 1,** which explains the various regimens of induction/enhancement of inflammation and USMI before and after the induction/enhancement of inflammation in mice.

### Chronic Colitis Models

#### IL-10^-/-^ Mice Treated with Piroxicam

*Group 1 and 2.* First, we evaluated the baseline inflammation with USMI in wild type C57BL/6 mice (group 1; n=5). For creating a chronic IBD model, we used IL-10^-/-^ mice (group 2; n=5; **Figure 1.2A**). The IL-10^-/-^ mice were switched from pelleted chow to powdered chow. Powdered chow was given for 7 days (day -14 to -7) to familiarize the mice with the changed form of food, and then 200 ppm piroxicam (Sigma-Aldrich, St. Louis, MO) was given in powdered chow for 7 days (day -7 to 0), as reported previously 22, 24, 27. After piroxicam was discontinued, the mice were given regular pelleted chow. At day 7, the IL-10^-/-^ mice were imaged with USMI, and euthanized immediately after the imaging session (see below; the details of imaging acquisitions are described in the section of Ultrasound Molecular Imaging) for *ex vivo* histology.

*Group 3.* In another group of IL-10^-/-^ mice (group 3; n=5; **Figure 1.2B**), the animals were given powdered chow for 7 days (day -14 to -7) and 200 ppm piroxicam in powdered chow for 7 days (day -7 to day 0). At the baseline before piroxicam was started, and then at days 7, 10 and 14, the mice were imaged with USMI, and euthanized after the last imaging session at day 14.

In the mice of groups 1, 2 and 3, middle colon was imaged with a clinical scanner, Acuson Sequoia 512 scanner (Siemens Healthcare, Malvern, PA). The details of imaging acquisitions are described in the Ultrasound Molecular Imaging section.

*Group 4.* In this group (n=5; **Figure 1.3A**), IL-10^-/-^ mice were given powdered chow for 7 days (day -14 to -7) and 200 ppm piroxicam in powdered chow for additional 7 days (day -7 to day 0). At the baseline before piroxicam was started and day 1, the mice were imaged with USMI, and euthanized after the last imaging session at day 1. From groups 4 to 15, the proximal, middle and distal colon of mice were imaged with a small animal scanner, Vevo 2100 (VisualSonics, Toronto, Canada), unless it was indicated that only the middle colon was imaged in some mice. The details of imaging acquisitions are described in the Ultrasound Molecular Imaging section.

*Group 5.* In this group (n=5; **Figure 1.3B**), IL-10^-/-^ mice were given powered chow for 7 days (day -7 to day 0) and 200 ppm piroxicam in powdered chow for 7 days (day -7 to day 0). At days 19, 26 and 33, the mice were imaged with USMI, and euthanized after the last imaging session for *ex vivo* analysis.

*Group 6.* In this group (n=5; **Figure 1.3C**), IL-10^-/-^ mice were given powered chow for 7 days (day -7 to day 0) and 200 ppm piroxicam in powdered chow for 35 days (day 0 to day 35). At days 7, 14, 21, 28, and 35, only the middle colon of mice was imaged with USMI, and the mice were euthanized after the last imaging session. 

#### IL-10^-/-^ Mice Treated with Ethanol/TNBS

*Group 7.* In this group (n=5; **Figure 1.4A**), 50% ethanol (EtOH) solution of 100 µL was administered intra-rectally at day 0. At the baseline (day -1) before EtOH was administered, and days 1, 7, 14, and 21, the mice were imaged with USMI, and euthanized after the last imaging session for *ex vivo* analysis.

*Group 8.* In this group (n=5; **Figure 1.4B**), the contact sensitizing agent TNBS (Sigma)/ EtOH solution (1:1, 100 µL in total) was administered rectally at day 0. At days 1, 10 and 21, the mice were imaged with USMI, and euthanized after the last imaging session for *ex vivo* analysis.

#### IL-10^-/-^ Mice Treated with DSS

*Group 9.* In this group (n=5; **Figure 1.5A**), 3% DSS (36,000-50,000 M.Wt.; MP Biomedicals; Santa Ana, CA) in the drinking water was used for 7 days (day -7 to 0). At days 1, 10, 21, and 28, the mice were imaged with USMI, and euthanized after the last imaging session.

*Group 10.* In this group (n=5; **Figure 1.5B**), 3% DSS in the drinking water was scheduled to administer for 21 days (day 0 to 21). At days 7 and 14, the mice were imaged with USMI, and all the mice died before the following imaging time point scheduled at day 21.

*Group 11.* In this group (n=5; **Figure 1.5C**), DSS in the drinking water was used for 16 days, and the dose of DSS was gradually lowered based on of body weight loss (2% DSS for day 0 to 9, 1.5% DSS for day 9 to 11; 1% DSS for day 11 to 16). At days 7, 14, 21, and 28, the mice were imaged with USMI, and euthanized after the last imaging session.

*Group 12.* In this group (n=5; **Figure 1.5D**), two repeated doses of 2% DSS in the drinking water (day 0-7, 15-21) separated by 1 week of normal drinking water (day 8-14, 22-28) were used. At days 7, 14, 21, and 28, the mice were imaged with USMI, and euthanized after the last imaging session.

#### FVB Mice Treated with DSS

*Group 13.* In this group (n=5; **Figure 1.6A**), 3% DSS in the drinking water was used for 21 days (day 0 to 21). At days 7 and 14, the mice were imaged using USMI, and all the animals died before the next scheduled imaging time point at day 21.

*Group 14.* In this group (n=5; **Figure 1.6B**), the dose of DSS was gradually reduced based on the general conditions and body weight loss of mice. 2% DSS in the drinking water was used for first 7 days (day 0 to 7), 1.8% DSS for following 7 days (day 7 to 14), and 1.5% DSS for last 14 days (day 14 to 28). At days 7, 14, 21 and 28, the mice were imaged using USMI, and euthanized after the last imaging session.

*Group 15.* In this group (n=6; **Figure 1.6C**), the dose of DSS was gradually reduced based on the general conditions and body weight loss of mice. 2% DSS in the drinking water was used for 7 days (day 0 to 7), 1.8% DSS for 7 days (day 7 to 14), and 1.5% DSS for 7 days (day 14 to 21). At the baseline before DSS was started, and days 7, 14 and 21, the mice were imaged using USMI, and euthanized after the last imaging session.

### Ultrasound Molecular Imaging

For the animals of group 1 to 3, USMI was performed with a clinical ultrasound scanner (Acuson Sequoia 512) and a clinical transducer (15L8 small footprint 28mm; center frequency, 8 MHz; lateral and axial resolution, 200 µm each; transmit power, -18 dB; mechanical index, 0.25; dynamic range, 80 dB). For animals from groups 4 to 15, a dedicated small animal ultrasound equipment (Vevo 2100) was used with a dedicated small animal transducer (MS250; center frequency of 18 MHz; lateral and axial resolution of 165 μm and 75 μm, respectively; transmit power, 10%; mechanical index, 0.2; dynamic range, 35 dB). Clinically translatable dual P- and E-selectin targeted contrast microbubbles (MB_Selectin_; Bracco Suisse SA, Geneva, Switzerland) were synthesized as previously described 17.

After mice were positioned on the ultrasound scanning table under gas anesthetized with 2% isoflurane in 2 L room air/min, the colon was first localized in B-mode at 2 cm distance from the anus using an electronic caliper available on the stage of the machine. Ultrasound molecular images were then acquired in the contrast mode at 4 min after an i.v. bolus injection of 1.25x10^7^ MB_Selectin_ for Acuson Sequoia 512 and 5x10^7^ MB_Selectin_ for Vevo 2100 via a catheter in the tail vein as described previously 19, 28. In brief, 10 seconds of imaging frames were first acquired, which detected signal from both molecularly attached targeted MBs and freely circulating MBs. This was followed by a 1-sec destruction pulse (transmit power, 100%; mechanical index, 1.9 for Acuson Sequoia 512 and 0.63 for Vevo 2100, respectively) to destroy all MBs in the same field of view. Immediately after the destruction pulse, another 10 seconds of imaging frames were acquired to obtain the signal from freely circulating MBs only.

The middle colon (2.0 cm from the anus) was imaged in all the mice. To show the discontinuous inflammatory lesions, the proximal (2.5 cm from the anus) and distal colon (1.5 cm from the anus) were also scanned in mice of groups 4, 5, and 7-15. To allow longitudinal monitoring of approximately the same locations of inflamed colon, the abdominal skin overlying the respective imaged locations was marked with a waterproof marker (Sharpie, Oak Brook, IL).

#### Analysis of Ultrasound Imaging Data Sets

All data sets were analyzed in random order by a reader with 7 years of experience in analyzing ultrasound images. A prototype software application (VueBox^®^; Bracco Suisse SA) with a dedicated module for USMI was used for the images acquired on Acuson Sequoia 512. A commercially available software (Vevo 2100) was used for the images acquired on Vevo 2100 scanner. For all the images, a region of interest (ROI) was drawn over the colon wall on all transverse images. The magnitude of USMI signal from the attached microbubbles was calculated as the difference between pre- and post-destructive pulse imaging intensity within the ROI in arbitrary units (a. u.).

### *Ex Vivo* Analysis of Colon Tissue

*Ex vivo* analysis of colonic tissue was performed in animals of groups 1-6 only. After imaging, animals were sacrificed, and previously imaged colon tissues were harvested. Tissues were fixed in 4% paraformaldehyde (PFA) at 4°C overnight and then preserved in a 30% sucrose solution at 4°C overnight. Samples were then placed in optimal cutting temperature compound (OCT) and frozen. From each frozen sample, 10 µm sections were obtained by using a cryomicrotome. Sections were incubated in phosphate buffered saline (PBS) for 10 min to remove the remaining OCT and permeabilized for 10 min in 0.5% Triton-X 100 in PBS. Sections were blocked in a solution containing 3% bovine serum albumin solution (Sigma), 3% goat serum (Sigma) and 3% donkey serum (Sigma) for 30 min at room temperature prior to incubation with primary antibodies [rat anti-mouse P-selectin (BD Biosciences, San Jose, CA) and rabbit anti-mouse CD31 (Abcam, Cambridge, MA)]. Primary antibodies were visualized with AlexaFluor488 donkey anti-rat IgG (Invitrogen, Grand Island, NY) and AlexaFluor 546 goat anti-rabbit IgG (Invitrogen). The F-actin cytoskeleton was visualized with AlexaFluor 633-phalloidin (Invitrogen). Samples were mounted in aqueous mounting media (BiogeneX, San Ramon, CA) and imaged using a 20X objective on a LSM510 metaconfocal microscope (Zeiss) using a 2X2 tiled confocal micrograph. Single confocal slices (1 µm) were collected and displayed.

For hematoxylin and eosin (H&E) staining, colonic tissues were cryosectioned into 10-μm-thick slices, and stained with H&E according to standard protocols 19. Sections were examined using an upright brightfield microscope (Olympus BX43; Tokyo, Japan). The criteria evaluated in the sections included evidence of fibrosis, edema, and neutrophilic inflammation. Fibrosis was defined as the relative increase in deposition of eosinophilic collagenous matrix leading to separation of the colonic gland profiles. Edema was defined as expansion of the tissue compartments by clear spaces or pale eosinophilic homogeneous glassy material (interpreted as fluid containing low and high protein content, respectively). Neutrophilic inflammation was defined as influx of neutrophils, either as discrete aggregates or scattered influx.

### Statistical Analysis

The paired Wilcoxon rank test was used to compare USMI signals with MB_Selectin_ of the same location (proximal, middle or distal colon) between different time points, respectively, whenever the data was paired. The two-sample Wilcoxon rank test was used to compare USMI signals between proximal, middle or distal colon at the same time point, or to compare USMI signals of the same location between different time points, when the data was not paired due to mortality of mice.

## Results

### General Conditions

**Table 2** summarizes the mortality of IL-10^-/-^ and FVB mice in all groups in response to the adaptation of different protocols used to enhance/induce inflammation in colon.

IL-10^-/-^ mice tolerated 200 ppm piroxicam for 7 days in groups 2, 3 and 4, while 20% (1/5) of mice died in group 5 at day 33. Exposure of the IL-10^-/-^ mice to 200 ppm piroxicam for 5 weeks was toxic; 20% (1/5) of mice in group 6 died at day 16, and only 40% (2/5) of mice in group 6 survived until day 35.

All the IL-10^-/-^ mice tolerated one-time administration of EtOH in group 7 and 100% (5/5) of mice survived until day 21. IL-10^-/-^ mice were more susceptible to one-time administration of TNBS/EtOH in group 8; 40% (2/5) of mice died at day 2.

IL-10^-/-^ mice tolerated 3% DSS for 7 days in group 9, and 5/5 mice survived through day 21. Administration of 3% DSS for 21 days was toxic to IL-10^-/-^ mice in group 10; mice started to die at day 15 (20%; 1/5 mice) and no mice (0/5) survived through day 21. When the dose of DSS was lowered (2% to 1%) based on the loss of body weight, the survival rate was improved: only 20% (1/5) of mice died at day 16 in group 11. When 2% DSS was used for the on-off cycle, all the mice (5/5 mice) in group 12 survived through day 28.

3% DSS for 21 days was toxic to FVB mice in group 13; mice started to die at day 15 (20%; 1/5 mice) and no mice (0/5 mice) survived through day 21. When the dose of DSS was lowered (2% to 1.5%) based on the loss of body weight, the survival rate was improved: 80% (4/5) of mice in group 14 survived through day 28; 33% (2/6) of mice in group 15 died at day 21 before the imaging session (**Table 2**).

### *In Vivo* USMI of Chronic Colitis in IL-10^-/-^ and FVB Mouse Models

**Figure 2:**
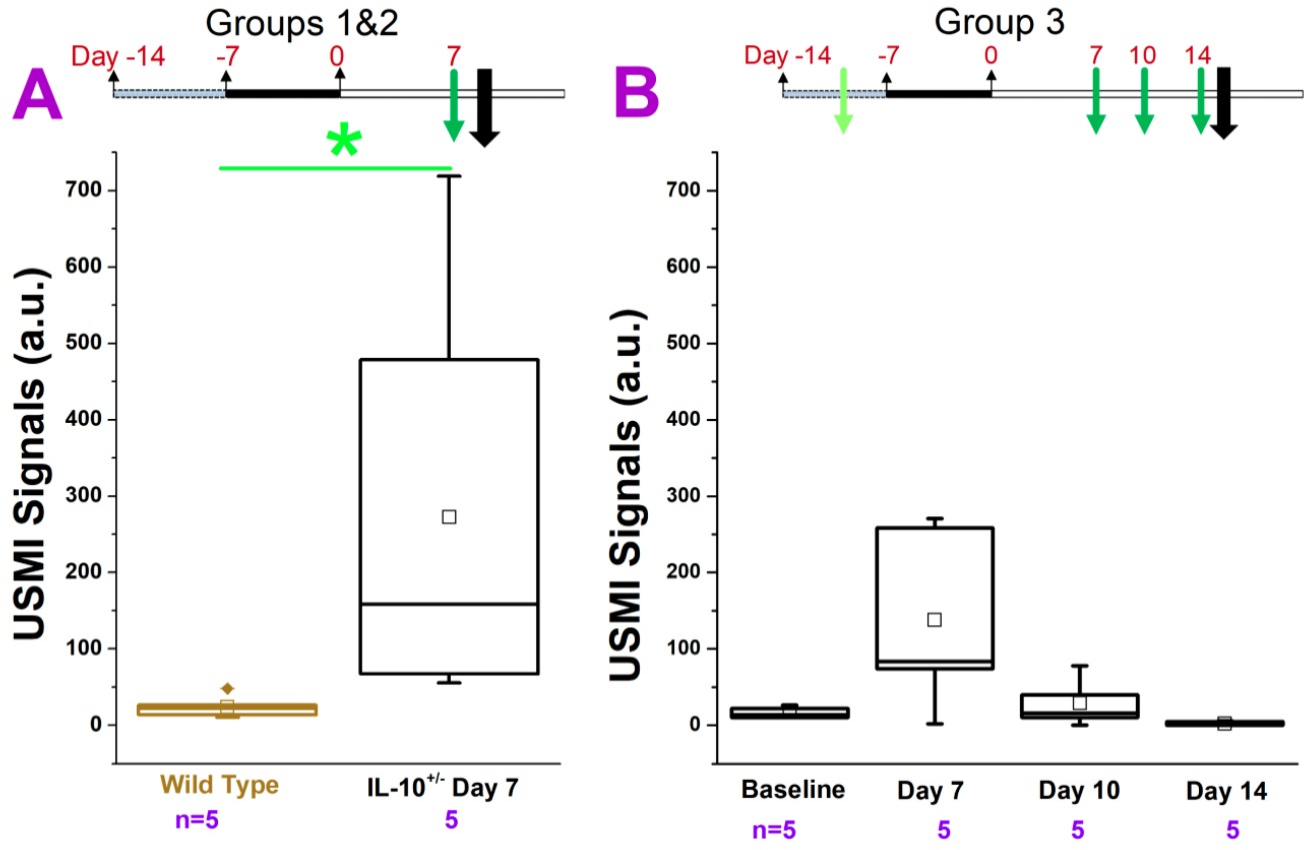
USMI signal intensity of IL-10^-/-^ mice treated with piroxicam and imaged using the Acuson Sequoia 512 scanner. **(A)** The USMI signals of middle colon in groups 1 and 2. **(B)** The USMI signals from the middle colon in group 3. Each box in the plot represents the 25^th^ and 75^th^ quartiles, the line inside each box identifies the median, and the small square indicates the mean. ♦ represents outliers.* indicates P value < 0.05 between the two boxes under the *.

**Figure 3:**
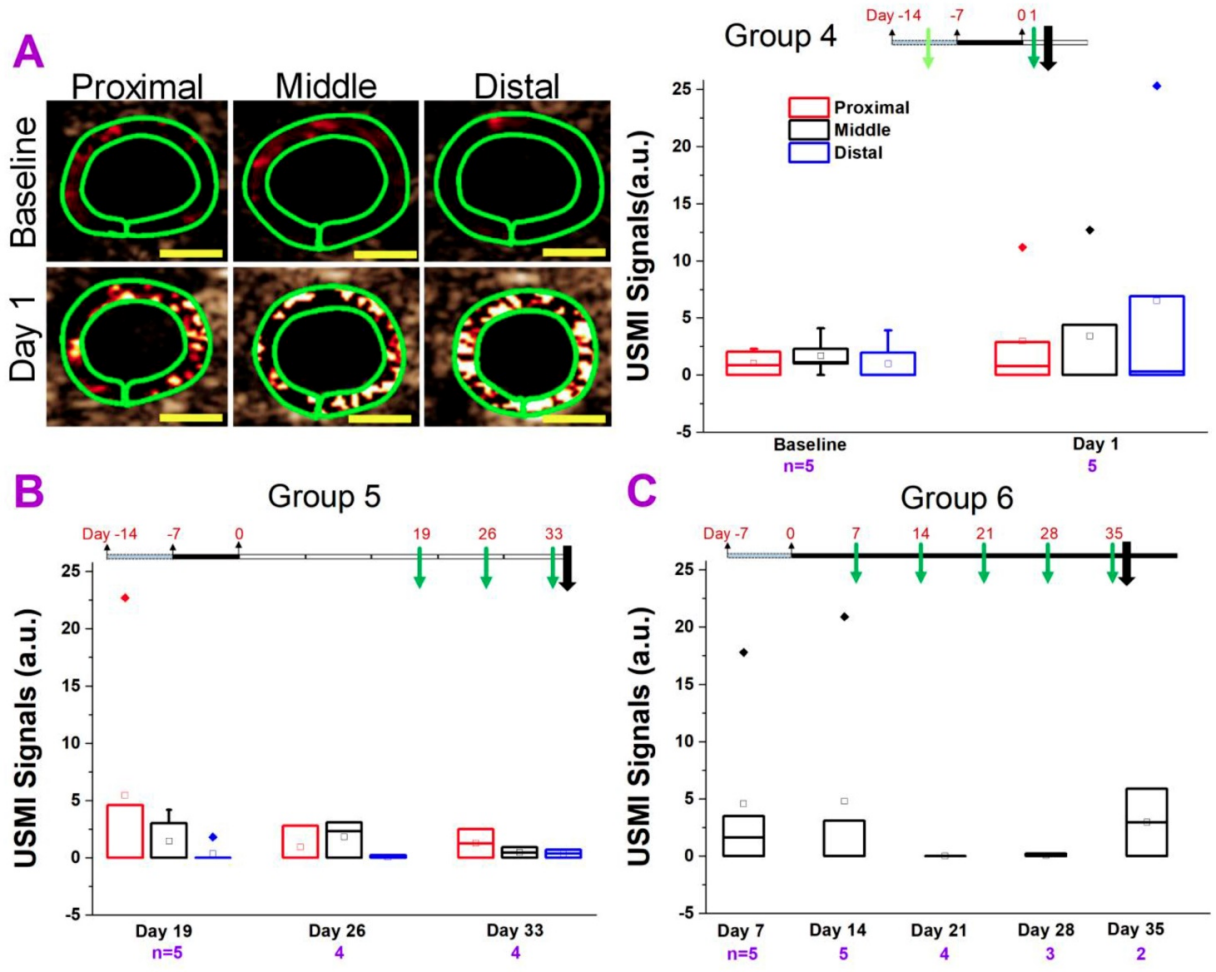
Comparison of USMI signals in different regions of the colon of IL-10^-/-^ mice treated with piroxicam and imaged with Vevo 2100 scanner. **(A)** Representative transverse ultrasound molecular images using Vevo 2100 scanner show the proximal (left column), middle (middle column) and distal colon (right column), in a mouse in group 4 at baseline level (before piroxicam was fed; top row) and at day 1 (bottom row) after piroxicam was discontinued. The imaging signals in proximal, middle and distal colon at day 1 are higher than at the baseline, but do not show the substantial locational differences. The green rings correspond to region of interest. Scale bars, 1 mm. The box chart summarizes the USMI signals from the proximal, middle or distal colon in group 4. **(B)** The USMI signals of proximal, middle and distal colon in group 5. **(C)** The USMI signals of middle colon in group 6.

**Figure 4:**
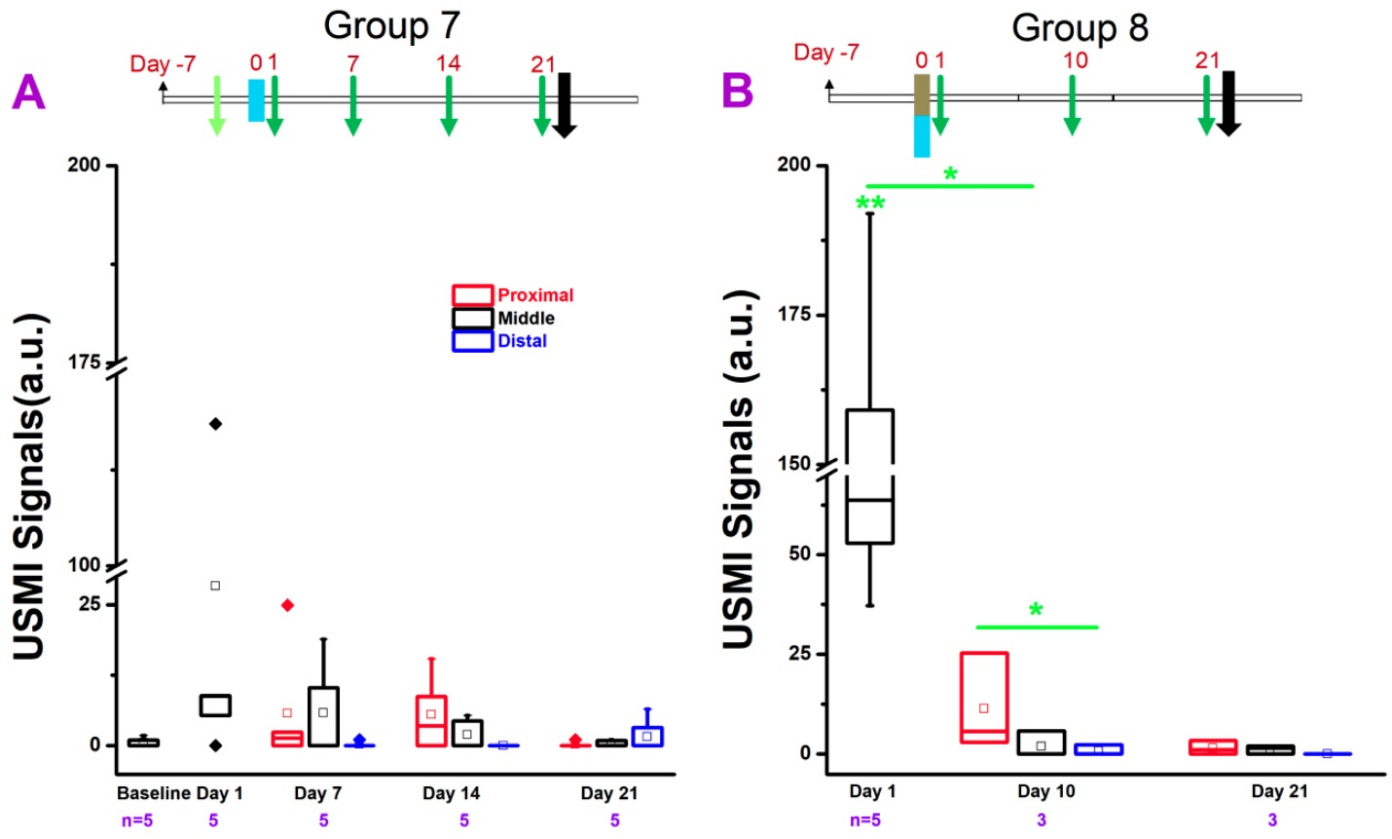
USMI signal intensity of IL-10^-/-^ mice induced with TNBS/EtOH, and imaged with the Vevo2100 scanner. **(A)** The USMI signals from the proximal, middle and distal colon from mice induced with EtOH in group 7. **(B)** The USMI signals from the proximal, middle and distal colon from mice induced with TNBS/EtOH in group 8. * indicates P value < 0.05 between the two boxes under the *. ** indicates P value < 0.05 between the box under the ** and the baseline value from the middle colon in group 7.

**Figure 5:**
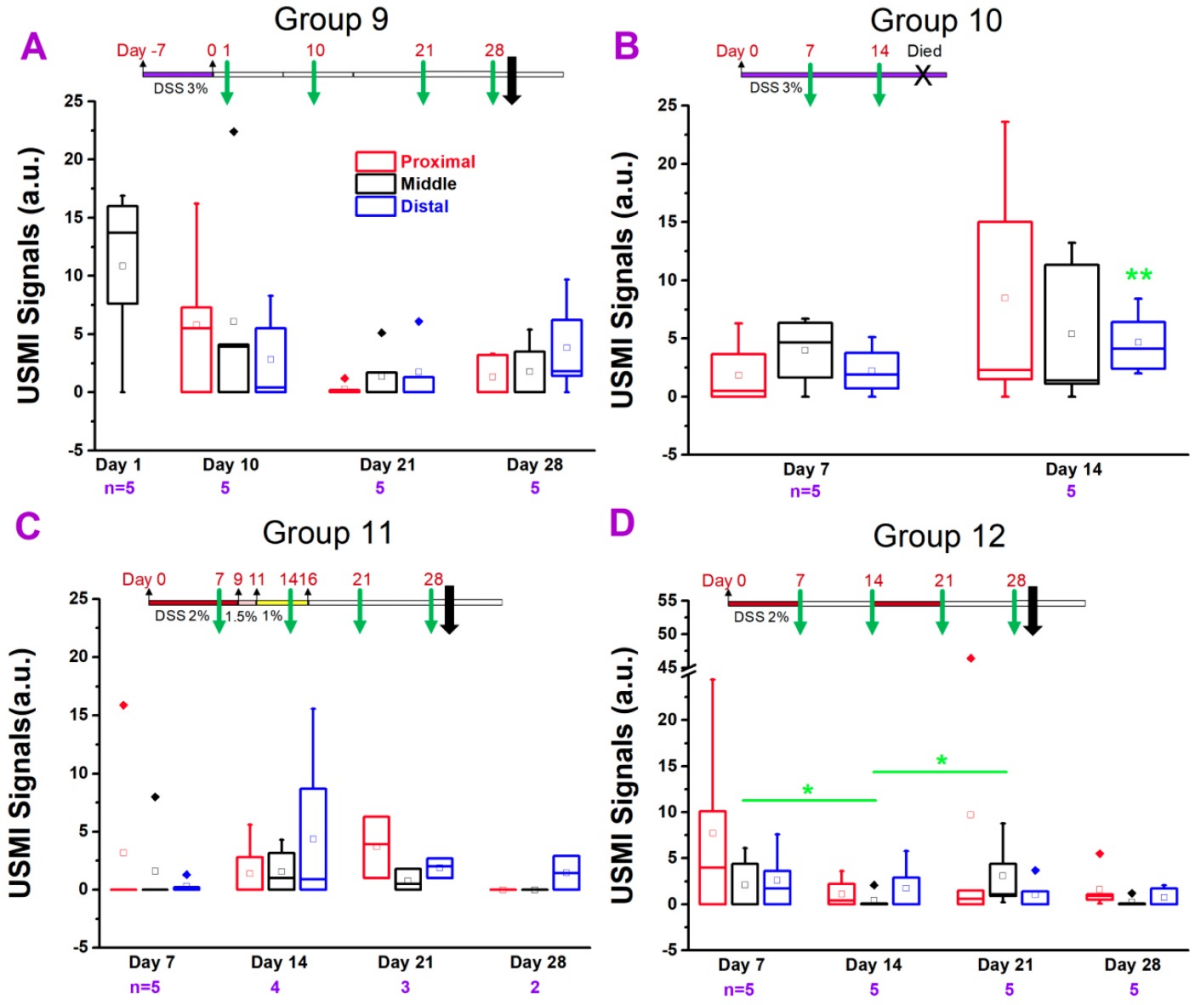
USMI signal intensity of IL-10^-/-^ mice induced with DSS and imaged using the Vevo2100 scanner. **(A)** The USMI signals from the proximal, middle or distal colon of mice induced with 3% DSS in group 9. **(B)** The USMI signals from the proximal, middle or distal colon of mice induced with 3% DSS in group 10. **(C)** The USMI signals from the proximal, middle or distal colon of mice induced with 2-1% DSS in group 11. **(D)** The USMI signals from the proximal, middle or distal colon of mice induced with 2% DSS on-off cycle in group 12. * indicates P value < 0.05 between the two boxes under the *. ** indicates P value < 0.05 between the box under the ** and the baseline value of distal colon in group 4.

**Figure 6:**
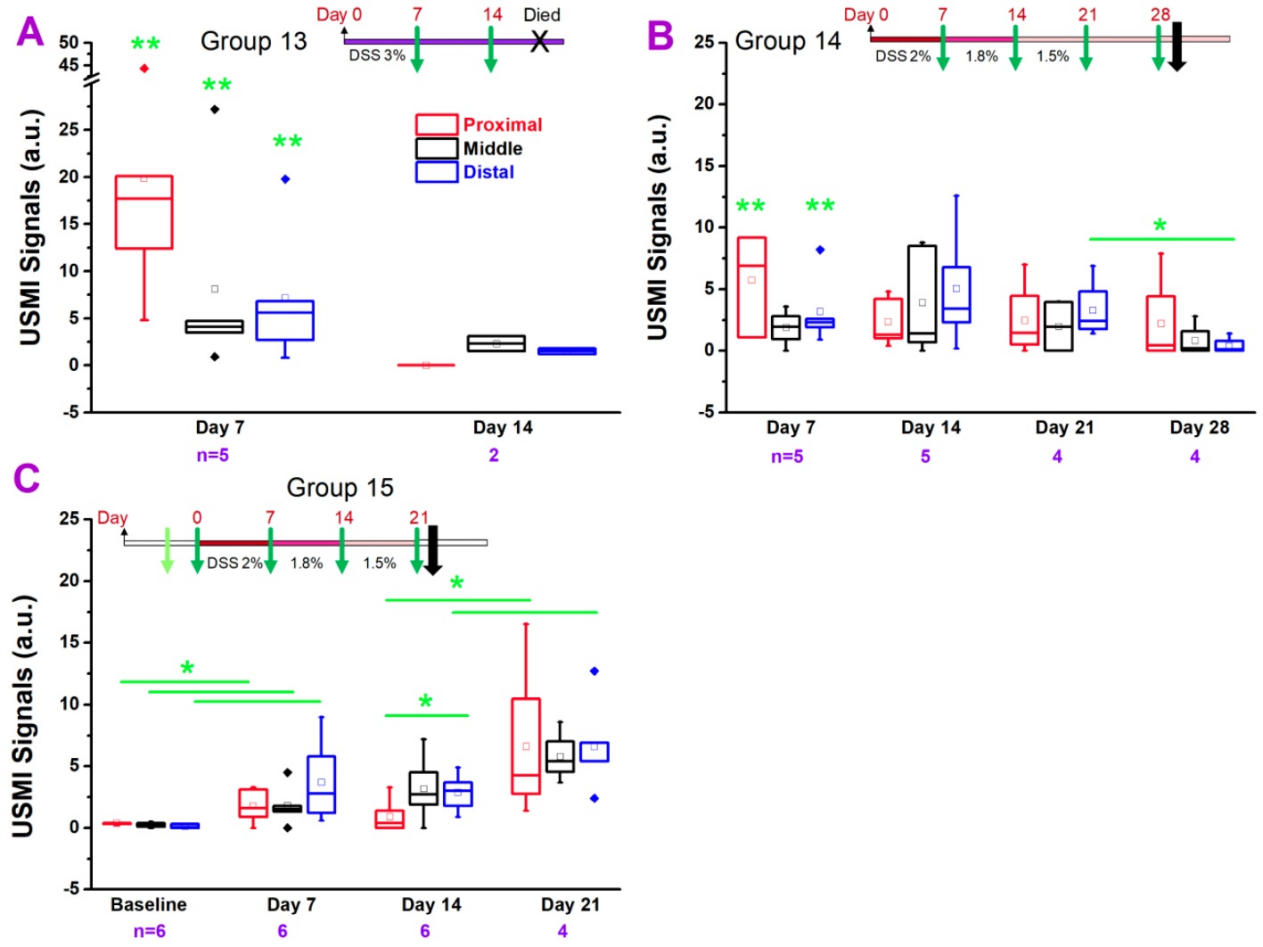
USMI signal intensity of FVB mice induced with DSS and imaged with the Vevo2100 scanner. **(A)** The USMI signals from the proximal, middle and distal colon of mice induced with 3% DSS in group 13. **(B)** The USMI signals from the proximal, middle and distal colon of mice induced with 2-1.5% DSS in group 14. **(C)** The USMI signals from the proximal, middle and distal colon of mice induced with 2-1.5% DSS in group 15. * indicates P value < 0.05 between the two boxes under the *. ** indicates P value < 0.05 between the box under the ** and the baseline values of the corresponding proximal, middle or distal colon in group 15.

We tested various models of chronic colitis in IL-10^-/-^ and FVB mice (induced with piroxicam, EtOH, TNBS, or DSS) with different dosage regimens, using clinical and small animal ultrasound scanners. The *in vivo* USMI results are summarized in **Figure 2-6**.

#### IL-10^-/-^ Mice Treated with Piroxicam and Imaged with Clinical US Scanner

In groups 1 and 2 **(Figure. 2A)**, we imaged the middle colon. The USMI signals were significantly (P = 0.01) higher (5 fold) in IL-10^-/-^ mice which received 200 ppm piroxicam for 7 days **(Figure 1.2A)** compared to wild type mice. Similarly, in group 3, we compared the USMI signals in the middle colon of IL-10^-/-^ mice at baseline (day 0) and at day 7 after piroxicam administration **(Figure 1.2B).** When compared to the baseline, USMI signals were higher at day 7 (P = 0.11), but the signal gradually dropped from day 7 to 11 and reached to the baseline level by day 14 **(Figure. 2B)**. There was no significant difference in the USMI signals between the 2 consecutive time points (P > 0.05 for baseline vs. day 7, day 7 vs. 11, and day 11 vs. 14).

#### IL-10^-/-^ Mice Treated with Piroxicam and Imaged with Small Animal US Scanner

In group 4 **(Figure 3A),** we used the same IBD model as shown in **Figure 2**. We used the small animal US scanner instead of the clinical scanner since the sensitivity of small animal scanner is much higher**.** We imaged the proximal, middle and distal colon segments. We imaged the mice at baseline and at day 1 only (just after piroxicam was discontinued) to determine the degree of inflammation. For all three segments (proximal, middle or distal), the USMI signals were not significantly (all P > 0.05) higher at day 1 after piroxicam was discontinued compared to baseline (before piroxicam), respectively **(Figure 1.3A, 3A)**. We also did not observe significant (all P > 0.05) locational difference between proximal, middle or distal colon at baseline or day 1 (**Figure 3A**).

In group 5 **(Figure 3B)**, we imaged the mice to determine if the inflammation would develop and persist as of 19 days after piroxicam was discontinued. For all three segments (proximal, middle or distal colon), the USMI signals were not significantly (P > 0.05) different at day 19 compared to baseline values in group 4 mice. The USMI signals of the proximal, middle or distal colon respectively were not significantly (all P > 0.05) different between the two consecutive time points of days 19 vs. 26, or 26 vs. 33 after piroxicam was discontinued **(Figure 1.3B)**. USMI signals did not show significant locational difference between the proximal, middle or distal colon at day 19, 26 and 33 (all P > 0.05).

In group 6 **(Figure 3C)**, piroxicam was continuously fed **(Figure 1. 3C)** from day 0 to 35. We imaged the mice for the evidence of inflammation from day 7 to 35. The USMI signal was not significantly (P = 0.15) different in middle colon at day 7 compared to baseline values in group 4 mice. The USMI signals of middle colon were not significantly (all P > 0.05) different between the any two consecutive time points of days 7, 14, 21, 28 and 35. We did not detect sustained inflammation in IL-10^-/-^ mice exposed to piroxicam with P- and E-selectin-targeted USMI using both clinical and small animal US scanners.

#### IL-10^-/-^ Mice Treated with TNBS/EtOH

TNBS/EtOH is frequently used for inducing acute colitis in mice. Ethanol compromises the mucosal barrier of the small bowel and TNBS is a haptenizing agent to induce a Th1-type inflammatory response 29, 30. In this study, we explored whether TNBS/EtOH or EtOH alone could induce sustained colitis in IL-10^-/-^ mice. In group 7 **(Figure 4A)**, the USMI signals were higher in the middle colon at day 1 compared to baseline before one-time EtOH administration (P = 0.07), and gradually decreased from day 1 to 21 without any further EtOH treatment **(Figure 1.4A)**. The USMI signals in the middle colon reached to the baseline level at day 21. The USMI signals were not significantly (all P > 0.05) different in middle colon between the any two consecutive time points of baseline, day 1, 7, 14, and 21 (all P > 0.05). At days 7, 14 and 21, USMI signals did not show significant locational difference between the proximal, middle or distal colon (all P > 0.05).

In group 8 **(Figure 4B)**, the USMI signals were significantly (P = 0.01) higher (168 fold) in the middle colon at day 1 after TNBS/EtOH **(Figure 1. 4B)** compared to baseline values of mice in group 7. The USMI signals in the middle colon significantly (P = 0.02) dropped from day 1 to day 10, and further decreased to baseline level at day 21. The USMI signal in the proximal colon was significantly (P = 0.046) higher than the distal colon at day 10. USMI signals did not show the significant locational difference between the proximal, middle or distal colon (all P > 0.05) at day 21. One-time administration of TNBS/EtOH induced a peak colitis in IL-10^-/-^ mice at day 1, but the inflammation was not sustained until day 10.

#### IL-10^-/-^ Mice Treated with DSS

DSS is one of the most frequently used chemicals for inducing colitis in mice 16, 31. In this study, we also tested whether DSS could induce a sustained colitis in IL-10^-/-^ mice. In group 9 **(Figure 5A)**, the USMI signals were higher (P = 0.09) in the middle colon at day 1 after 3% DSS was administered for 7 days (from day -7 to 0) **(Figure 1.5A)** compared to baseline values in group 4 mice, and the signals gradually decreased from day 1 to 28. The USMI signals were not significantly different in middle colon between the two consecutive imaging time points of days 1, 10, 21, and 28 (all P > 0.05). At days 10, 21 and 28, there was no significant locational difference in the USMI signals between the proximal, middle or distal colon (all P > 0.05).

In group 10 **(Figure 5B)**, we exposed the mice to 3% DSS continuously. The USMI signals were higher in proximal, middle and distal colon at day 7 after continuous 3% DSS (started on day 0) **(Figure 1.5B)** compared to baseline values in group 4 mice, respectively (all P > 0.05), and further increased from day 7 to 14 (all P > 0.05). When the USMI signals at day 7 or 14 were compared to the baseline values of group 4 in proximal, middle and distal colon, respectively, only the USMI signals of the distal colon at day 14 were significantly (P = 0.046) higher than the baseline. There was no significant locational difference in the USMI signals between the proximal, middle or distal colon at days 7 and 14 (all P > 0.05). Although the USMI signals gradually increased from day 7 to 14 with continuously administered 3% DSS, no mice survived through the next scheduled imaging session (day 21).

In group 11 **(Figure 5C)**, the USMI signals were higher in the proximal, middle and distal colon at day 7 after continuous 2% DSS since day 0 **(Figure 1. 5C)** compared to baseline values in group 4 mice (P >0.05), respectively, and further increased from day 7 to 14 (P >0.05), then dropped on 28 (all P > 0.05). The USMI signals were not significantly (all P > 0.05) different in the proximal, middle or distal colon between the 2 consecutive time points (all P > 0.05). There was no significant locational difference in the USMI signals from the proximal, middle or distal colon at days 7, 14, 21, and 28 (all P > 0.05).

In group 12 **(Figure 5D)**, the USMI signals were higher in the proximal, middle and distal colon at day 7 after 2% DSS (on for days 0-7 and 14-21; off for days 7-14 and 21-28; **Figure 1.5D**) compared to baseline values in group 4 mice (all P > 0.05), respectively, then dropped from day 7 to 14. The USMI signals were not significantly (all P > 0.05) different in proximal, middle or distal colon between the two consecutive time points (P > 0.05) except for the middle colon (P = 0.045 for day 7 vs. 14, and for day 14 vs. 21). At day 7, 14, 21, and 28, USMI signals did not show the significant locational difference between proximal, middle or distal colon (all P > 0.05). The inflammation in IL-10^-/-^ mice was not enhanced and sustained by using ≤3% DSS with an acceptable mortality rate.

#### FVB Mice Treated with DSS

We also explored the feasibility of inducing chronic colitis with DSS in FVB mice (groups 13 to 15).

In group 13 **(Figure 6A)**, the USMI signals were significantly higher (all P = 0.01) in the proximal, middle and distal colon at day 7 after continuous treatment of 3% DSS **(Figure 1.6A)** compared to baseline values in group 15 mice, respectively, and the signals dropped from day 7 to 14 (all P > 0.05). None of the mice survived until the imaging session scheduled for day 21. At day 7 and 14, USMI signals did not show the significant locational difference between the proximal, middle or distal colon (all P > 0.05).

In group 14 **(Figure 6B)**, the USMI signals were higher in the proximal (P = 0.02), middle (P = 0.16) and distal (P = 0.01) colon at day 7 after continuous DSS (starting at 2% and gradually lowered dose based on the loss of body weight, **Figure 1.6B**) compared to baseline values in group 15 mice, respectively, and fluctuated at days 14 and 21 (all P > 0.05). The USMI signals dropped in the proximal, middle and distal (P = 0.03) colon from day 21 to 28. There was no significant locational difference in the USMI signals from the proximal, middle or distal colon at days 7, 14, 21 and 28 (all P > 0.05).

In group 15 **(Figure 6C)**, the USMI signals were significantly higher in proximal (P = 0.046), middle (P = 0.04) and distal colon (P = 0.03) at day 7 after continuous DSS (starting at 2% and gradually lowered the dose based on the loss of body weight, **Figure 1.6C**) compared to baseline values, respectively, and fluctuated from day 7 and 14 (all P > 0.05). The USMI signals increased in the proximal (P = 0.02), middle (P = 0.09) and distal (P = 0.04) colon from day 14 to 21, with a mortality rate of 33% until day 21. There was no significant locational difference in the USMI signals between the proximal, middle or distal colon at day 7, 14, and 21, except for proximal vs. distal colon at day 14; P=0.03). The colitis in FVB mice was induced with DSS, but not sustained by using ≤3% DSS with an acceptable mortality rate.

We explored the use of piroxicam, EtOH, TNBS, and DSS with various dosage regimens for inducing/enhancing chronic colitis in both IL-10^-/-^ and FVB mice, and monitored the chronic colitis with dual-selectin targeted USMI by using clinical and small animal US scanners. Even though many of dosage regimens were able to induce inflammation, none of these models showed sustained colitis for up to 14-35 days (which is needed for long-term monitoring of drug mediated treatment effect) with substantially higher USMI signals compared to the baseline conditions. In addition, many of these regimens also induced significant mortality in animals.

### *Ex Vivo* Analysis

#### Immunofluorescence Staining

**Figure 7:**
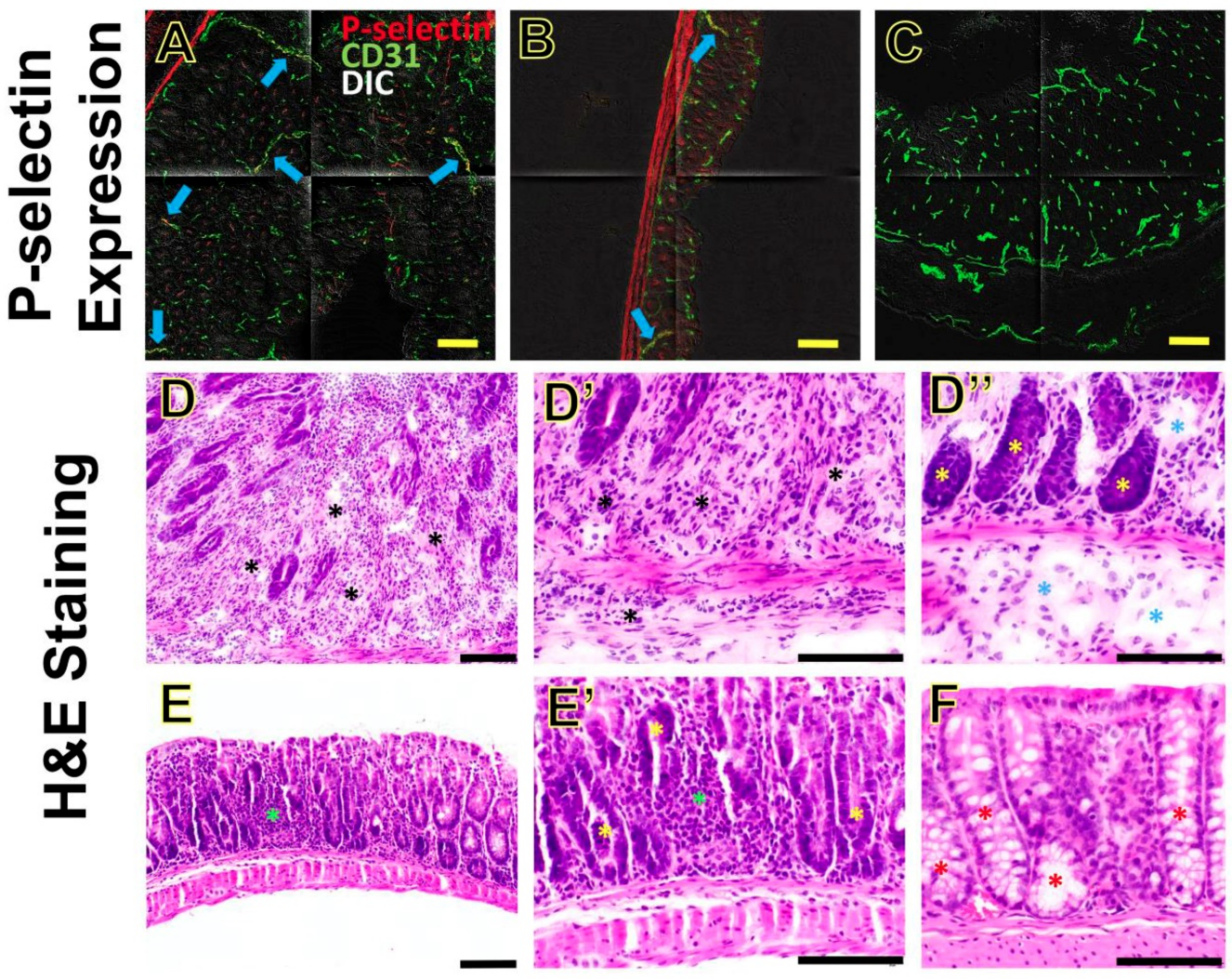
P-selectin expression and inflammation in section of colon from mice induced with piroxicam. **(A-B)** Immunofluorescence shows moderate **(A)** or mild **(B)** expression (blue arrows) of P-selectin (highlighted in red) on CD31-positive vascular endothelial cells (highlighted in green) in the inflamed colon of two separate IL-10^-/-^ mice **(A and B, respectively),** both at day 35 after piroxicam was discontinued. **(C)** Background expression of P-selectin in the colon of a wild type mouse. Tiled confocal micrographs were overlaid on differential interference contrast (DIC) images. **(D-F)** Hematoxylin and eosin (H&E)-stained sections of colon from different treatment groups show differences in the degree of inflammation. Sections from an IL-10^-/-^ mouse (tissue collected at at day 33 after piroxicam was discontinued, Cohort A) **(D, D', D'')** show evidence of neutrophilic inflammation that sometimes leads to significant expansion of the lamina propria and separation of the colonic glands (**D-D'**, black asterisks). Gland profiles are separated, and the submucosa are often expanded, by edema fluid (**D''**, blue asterisks). H&E-stained sections of colon (**E, E'**) from another IL-10^-/-^ mouse (tissue collected at at day 7 after piroxicam was discontinued, Cohort B) show scattered neutrophilic inflammation that is generally limited to the lamina propria (green asterisks), and lacks evidence of edema or fibrosis. In sections from both Cohort A and Cohort B, the epithelial lining of the colonic glands lacks overt cell differentiation (**D'', E'**, yellow asterisks), in contrast to sections of the Wild Type Cohort (**F**, red asterisks). Sections from the Wild Type Cohort (**F**) lack neutrophilic inflammation, edema, or fibrosis. Scale bars, 100 µm.

P-selectin expression was assessed in the IL-10^-/-^ mice from 1-35 days after piroxicam was discontinued (**Figure 7A, B**) and wild type mice **(Figure 7C).** There was only background expression of P-selectin in wild type mice of group 1 **(Figure 7C)**. In the IL-10^-/-^ mice of groups 2-6, the colon tissues were harvested for immunofluorescence staining from 1-35 days after piroxicam was discontinued. Mild to moderate expression of P-selectin was observed, but no substantial difference was shown between different groups of animals treated with piroxicam, in which immunofluorescence staining was performed at days 1, 7, 14, 33, or 35 after piroxicam was discontinued **(Figure 7A, B)**.

#### H&E Staining

The colon tissues were categorized into Cohort A (groups 3, 5 and 6; the colon tissues were harvested at day 14, 33 and 35 after piroxicam was discontinued), Cohort B (groups 2 and 4; the colon tissues were harvested at day 1 or 7 after piroxicam was discontinued), and Wild Type Cohort (group 1). We chose the three cohorts reflecting various days after piroxicam was discontinued to show whether the inflammatory changes on H&E staining were progressive or not.

Overall, the sections from Cohort A had the most profound changes, compared to sections from Cohort B and Wild Type Cohort **(Figure 7D-F)**. In the sections from Cohort A (day 14, 33 or 35), neutrophilic inflammation was usually present in the lamina propria, sometimes extending to the submucosa and less frequently to the muscular layer **(Figure 7D, D', D'')**. Neutrophilic inflammation in Cohort A generally spanned a large region, and sometimes was close to being diffuse. In addition, sections from Cohort A often exhibited edema and some degree of fibrosis in the lamina propria **(Figure 7D'')**. Tissue sections from Cohort B (day 1 or 7) had significantly less inflammation than those of Cohort A, and when present it was multifocal and typically limited to lamina propria and rarely extending to the submucosa **(Figure 7E, E')**. In the sections from Cohort B, there was no evidence of edema or fibrosis. In both Cohort A and B, the epithelial lining of the colonic glands often lacked goblet cell differentiation **(Figure 7D'', 7E')**, in contrast to sections from the Wild Type Cohort **(Figure 7F)** in which goblet cell differentiation was evident. Tissue sections from the Wild Type Cohort had no evidence of ulceration, fibrosis, edema, or neutrophilic inflammation **(Figure 7F)**. These H&E findings indicate that the inflammation was progressing from day 1-7 (Cohort B) to days 14-35 (Cohort A) after piroxicam was discontinued.

## Discussion

This study shows the results of our extensive attempts to develop chronic IBD models that can maintain inflammation for an extended period and mimic human IBD, and that can be assessed on USMI. We used IL-10^-/-^ mice (which have been extensively studied for IBD pathomechanism in the literature), and FVB mice. We observed a similar disease phenotype as in human IBD where P-selectin is overexpressed (for which targeted USMI can be employed). However, sustained colitis in various chronic models using IL-10^-/-^ and FVB mice was not detected with USMI using dual-selectin targeted microbubbles in longitudinal follow-up imaging exams. Thus, these chronic models of colitis may not be suitable for long-term longitudinal monitoring of anti-inflammatory treatment response by using dual-selectin targeted USMI.

Transabdominal ultrasound is a well-established imaging tool for the assessment of patients with IBD. USMI with MB_Selectin_ was shown to quantify inflammation at a molecular level in small and large animal models of IBD and is thus a promising tool for long-term monitoring of the disease activity. So far, USMI has been used to diagnose the disease and monitor the treatment response in rodent and swine models of acute or acute on chronic IBD 15-18.

Mouse IBD models with acute chemically induced inflammation are extensively used for studying mechanisms of IBD and treatment monitoring 32. Among them, IBD models induced by DSS and TNBS are most widely used, because of their simplicity to induce, low cost, rapid onset of inflammation, and histopathological resemblance to human IBD 32. However, human IBD is a naturally occurring disease with chronicity, and is often of life-long duration. These acute IBD models fail to recapitulate the spontaneous onset and chronicity of IBD, and are not suitable for long-term monitoring of treatment outcomes. Therefore, IBD models with spontaneous development of inflammation are warranted.

The IL-10^-/-^ mouse model is one of the most frequently used models of chronic IBD and has been extensively studied for almost three decades 20, 23, 33, 34. IL-10 is an anti-inflammatory cytokine and suppresses production of pro-inflammatory cytokines. Although IL-10^-/-^ mice were originally reported to spontaneously develop colitis, it has been shown that IL-10^-/-^ mice are resistant to the development of spontaneous colitis when kept in pathogen free environment. When exposed to non-steroidal anti-inflammatory drugs (NSAIDs) such as piroxicam, IL-10^-/-^ mice can develop rapid (< 2 weeks) and uniform colitis, which sustains for a long time after piroxicam is discontinued, as verified on H&E staining 22, 24. However, P-selectin expression level was not determined in those studies.

In the current study, we first exposed IL-10^-/-^ mice to piroxicam in powdered chow for a week and started to image the mice with dual-selectin targeted USMI from day 7 to 14 after piroxicam was discontinued. To be more clinically translatable, we used a clinical ultrasound scanner (Acuson Sequoia 512) with room air for gas anesthesia. We found out that the UMSI signals from the middle colon in IL-10^-/-^ mice in group 2 at day 7 were significantly higher than wild type mice. When we imaged the mice in group 3 at day 7, 10 and 14, the USMI signals at day 7 were higher than the baseline value before piroxicam, but UMSI signals did not sustain from day 7 through 14.

Since we did not observe sustained inflammation in IL-10^-/-^ mice induced with piroxicam by using the clinical US scanner, we switched to the small animal dedicated US scanner with higher imaging resolution in the subsequent groups of animals. In addition, we started to image proximal, middle and distal colonic segments to detect whether there were locational differences in the degree of inflammation throughout the colon of mice. USMI signals from the proximal, middle and distal colon after a 7-day exposure of piroxicam were all higher at day 1 than the baseline values, but the signals gradually decreased from day 1 to day 33. No substantial locational differences between the proximal, middle and distal colon were observed in any imaging acquisitions. According to unpublished data from other groups (Dr. Laura Hale), IL-10^-/-^ mice start to show persistent colitis at day 19 after piroxicam is used in the diet for one week as verified on H&E staining. However, we did not observe the same outcome by using USMI. In group 6, we also exposed the mice to piroxicam for up to 35 days. The USMI signals from the middle colon as of day 7 were not significantly higher than baseline value, and the USMI signals at day 35 were not significantly higher than day 7, even with continuous administration of piroxicam for 35 days. In addition, mortality rate was high as of day 21.

Since piroxicam was not effective in enhancing the development of sustained chronic colitis to a level which could be detected with USMI, we also tested the feasibility of TNBS at inducing sustained chronic colitis in IL-10^-/-^ mice. TNBS is one of most frequently used chemicals for induce acute colitis in BALB/c mice 29, 30. TNBS haptenizes the microbiota in intestine and thus induces a Th1-type response in the murine colon 29, 35. Our hypothesis was that one TNBS dose could induce acute onset of colitis and the acute inflammation could be sustained and progress to chronic colitis in IL-10^-/-^ mice. In group 8, TNBS/EtOH did induce a peak inflammatory process at day 1, but the inflammation rapidly subsided by day 10. In addition, IL-10^-/-^ mice drastically responded to TNBS, and mortality rate was high by day 1 of treatment. Our experiences show that repeated doses of TNBS/EtOH substantially increase the mortality rate of BALB/c mice (unpublished data). Therefore, we did not try repeated does of TNBS in the current study.

DSS is another frequently used chemical for inducing chronic IBD 16, 36. DSS has a direct toxic effect on the intestinal epithelial cells, and the integrity of the mucosal barrier is compromised with DSS, leading to the development of inflammation 37-39. In the current study, we tested various dosages of DSS (ranging from 3% to 1%) and various durations of treatment (ranging from 7 days, on-off cycle (7 days each) and up to 21 days). Only the IL-10^-/-^ mice treated with 3% DSS showed gradually increasing USMI signals from baseline levels to day 14. However, this dose of 3% DSS was indeed toxic to the mice and all the mice died before day 21. IL-10^-/-^ mice treated with less than 3% DSS and/or for less than 14 days did fail to have sustained inflammation as assessed by USMI.

FVB mice are frequently used mouse strain for studying chronic IBD models induced with DSS 16, 31. In the current study, we tested whether a chronic IBD model could be established in FVB mice that would allow for using USMI as a diagnostic application. Our results showed that FVB mice were not tolerant to 3% DSS for up to 21 days. In addition, USMI signals substantially dropped from day 7 to day 14 as seen in the IL-10^-/-^ model. In other FVB mouse groups where we treated with less than 2% DSS, sustained inflammation could not be detected by USMI, even shortly after induction.

In the current study, we started with IL-10^-/-^ models of chronic colitis induced with piroxicam. We tested various durations of piroxicam exposure, multiple time points for imaging acquisition and also queried locational differences between the proximal, middle and distal colon. In some groups, we observed increased USMI signal at early time points after induction of colitis using piroxicam. However, we did not detect sustained colitis with dual-selectin targeted USMI. Then we proceeded with IL-10^-/-^ models in which colitis was induced with TNBS or DSS, and eventually, FVB mice induced with DSS. Our results show that genetically engineered mice such as IL-10^-/-^ mice, although prone to developing spontaneous colitis, failed to develop sustained chronic colitis with piroxicam promotion for dual-selectin targeted USMI purposes. Progressive inflammatory changes were observed on H&E staining of IL-10^-/-^ mice from day 1 to 35 after piroxicam was discontinued, which is in agreement with the findings by Hale et al 22, 24. However, stable overexpression of P-selectin was not observed on immunofluorescence staining in IL-10^-/-^ mice at multiple time points after piroxicam was discontinued. Our previous studies showed that *in vivo* dual-selectin targeted USMI signals correlate with *ex vivo* overexpression of P-selectin 15-18. This explains why sustained colitis was not detected with dual-selectin targeted USMI at multiple time points after piroxicam was discontinued. Other chemicals such as TNBS or DSS could induce colitis in IL-10^-/-^ or FVB mice. However, both failed to establish colitis that was maintained over time, and the mortality rate was not acceptable in these mouse models. Our goal was to establish a chronic IBD model where USMI signals are maintained moderate/high up to 3 weeks and P-selectin is stably overexpressed for assessing anti-inflammatory treatment effect. Unfortunately, none of the tested models in this study fulfills such requirements.

It was previously shown that MAdCAM-1 targeted USMI could quantify the terminal ileitis in a transgenic model of IBD 26. Spontaneous ileitis in SAMP model was quantified with USMI and verified on *ex vivo* histology. IBD lesions in SAMP model develop by 20 weeks of age with 100% penetrance. The IBD lesions in SAMP model are mainly localized to terminal ileum 40. It is challenging to image the terminal ileum due to its anatomical location in abdominal cavity, especially when the exact same region of terminal ileum needs to be imaged during the multiple longitudinal follow-up exams for monitoring the evolution of disease activity. Therefore, we chose to test the IBD models with lesions in colon region in the current study, which is relatively easy to image compared to terminal ileum. Dual P and E-selectin-targeted USMI has been shown to quantify IBD and monitor the treatment effects in acute models of IBD, while this imaging approach needs to be explored in more chronic IBD models other than the ones tested in the current study.

We also acknowledge the following study limitations. Firstly, we tested various mouse models of chronic colitis by using different experimental protocols, while the sample size for each group was small. Moreover, the mortality rate in some groups was high, which resulted in a less robust statistical analysis. However, our results show that even though the sample size was limited, it was clear that the sustained colitis was not detected in any of the animal groups on USMI. Therefore, we decided not to repeat these animal experimental protocols in a large-scale. Secondly, we did not perform the *ex vivo* analysis in all animals. We did not observe stable overexpression of P-selectin in colonic tissues of IL-10^-/-^ mice in group 2-6 on immunofluorescence staining at multiple time points after piroxicam was discontinued. This *ex vivo* finding was accordant with *in vivo* dual-selectin targeted USMI signals in the current study. In addition, our previous studies also confirmed the correlation between *in vivo* dual-selectin targeted USMI signal and *ex vivo* P-selectin expression 15-18. Furthermore, assessment of H&E-stained sections showed that inflammatory changes were found in colonic tissues of IL-10^-/-^ mice as of 19 days after piroxicam was discontinued, which was in agreement with the studies by Hale et al 22, 24. Based on these, we did not perform *ex vivo* analyses in the other groups of animals. Thirdly, we did not perform 3-dimensional USMI in the current study. To overcome the sampling limitation of 2-dimensional USMI, we imaged the proximal, middle and distal colon of the mice, and our results did not show the locational differences in the degree or severity of colitis in IL-10^-/-^ and FVB mice. In the future studies, 3-dimensional USMI is warranted for overall evaluation of the inflammatory conditions in the colon of mice. Lastly, we did not test other chronic IBD models, e.g. SAMP model of terminal ileitis, or other molecular markers which are stably expressed in chronic IBD, such as MAdCAM-1^41^, ^42^. In the future studies, USMI with more potential molecular markers need to be explored in more chronic IBD models.

In conclusion, our results show that sustained colitis in IL-10^-/-^ mice induced with piroxicam, TNBS or DSS, and in FVB mice induced with DSS was not detected with ultrasound molecular imaging using P- and E-selectin targeted microbubbles. Thus, these IBD models may not be appropriate for the long-term monitoring of chronic colitis and assessment of treatment response with dual-selectin targeted ultrasound molecular imaging.

## Figures and Tables

**Table 1:** Regimens of Induction/enhancement of Colitis in IL-10^-/-^ and FVB Mice with Different Drug/agents

	Model	Drug/agents		Treatment Regimen
1	IL-10^-/-^	Piroxicam		Continuously for 7 days
2	IL-10^-/-^	Piroxicam		Continuously for 35 days
3	IL-10^-/-^	EtOH		Single dose
4	IL-10^-/-^	TNBS+EtOH		Single dose
5	IL-10^-/-^	3% DSS		Continuously for 7 days
6	IL-10^-/-^	3% DSS		Continuously for 21 days
7	IL-10^-/-^	2%, 1.5%, 1% DSS		Continuously for 16 days; gradually lowered doses based on loss of body weight
8	IL-10^-/-^	2% DSS		On-off cycle: on for days 0-7; 15-21; off for days 8-14, 22-28
9	FVB	3% DSS		Continuously for 21 days
10	FVB	2%, 1.8%, 1.5% DSS		Continuously for 28 days: gradually lowered doses based on loss of body weight
11	FVB	2%, 1.8%, 1.5% DSS		Continuously for 21 days: gradually lowered doses based on loss of body weight

TNBS: 2,4,6-trinitrobenzenesulfonic acid; EtOH: ethanol; DSS: dextran sulfate sodium.

**Table 2:** Mortality of IL-10^-/-^ and FVB Mice after Administration of Drugs and Chemicals.

Group	Model	Drug/agents	Treatment Duration	Earliest Mortality	Mortality at Last Time Point
1	Wild type	NA	NA	NA	NA
2	IL-10^-/-^	Piroxicam	Day -7 to 0	NA	Day 7 (0/5)
3	IL-10^-/-^	Piroxicam	Day -7 to 0	NA	Day 14 (0/5)
4	IL-10^-/-^	Piroxicam	Day -7 to 0	NA	Day 1 (0/5)
5	IL-10^-/-^	Piroxicam	Day -7 to 0	Day 32 (1/5)	Day 33 (1/5)
6	IL-10^-/-^	Piroxicam	Day 0 to 35	Day 16 (1/5)	Day 35 (3/5)
7	IL-10^-/-^	EtOH	Once	NA	Day 21 (0/5)
8	IL-10^-/-^	TNBS+EtOH	Once	Day 1 (2/5)	Day 21 (2/5)
9	IL-10^-/-^	3% DSS	Day -7 to 0	NA	Day 21 (0/5)
10	IL-10^-/-^	3% DSS	Day 0 to 21	Day 15 (1/5)	Day 21 (5/5)
11	IL-10^-/-^	2%, 1.5%, 1% DSS	Day 0 to 16	Day 16 (1/5)	Day 21 (3/5)
12	IL-10^-/-^	2% DSS	Day 0-7; 14-21	NA	Day 28 (0/5)
13	FVB	3% DSS	Day 0 to 21	Day 15 (1/5)	Day 21 (5/5)
14	FVB	2%, 1.8%, 1.5% DSS	Day 0 to 28	Day 18 (1/5)	Day 28 (1/5)
15	FVB	2%, 1.8%, 1.5% DSS	Day 0 to 21	NA	Day 21 (2/6)

Earliest mortality indicates the time point when the mice started to die (the number of mice is indicated in parentheses). Mortality at last time point indicates the number of mice that had not survived through the last scheduled time point for imaging acquisition.

## Abbreviations

a.u.: arbitrary unit
CEUS: contrast-enhanced ultrasound
DSS: dextran sulfate sodium
EtOH: ethanol
H&E: hematoxylin and eosin
IBD: inflammatory bowel disease
IL-10^-/-^: Interleukin 10 deficient
MB_Selectin_: dual P- and E-selectin targeted microbubbles
PBS: phosphate buffered saline
PFA: paraformaldehyde
OCT: optimal cutting temperature compound
ROI: region of interest
TNBS: 2,4,6-trinitrobenzenesulfonic acid
USMI: ultrasound molecular imaging
